# Proteomic Profiling and T Cell Receptor Usage of Abacavir Susceptible Subjects

**DOI:** 10.3390/biomedicines10030693

**Published:** 2022-03-17

**Authors:** Eline Gall, Florian Stieglitz, Andreas Pich, Georg Martin Norbert Behrens, Joachim Kuhn, Rainer Blasczyk, Funmilola Josephine Haukamp, Christina Bade-Döding

**Affiliations:** 1 Institute for Transfusion Medicine and Transplantat Engineering, Hannover Medical School, Carl-Neuberg-Str. 1, 30625 Hannover, Germany; gall.eline@mh-hannover.de (E.G.); blasczyk.rainer@mh-hannover.de (R.B.); bade-doeding.christina@mh-hannover.de (C.B.-D.); 2 Institute of Toxicology, Hannover Medical School, Carl-Neuberg-Str. 1, 30625 Hannover, Germany; stieglitz.florian@mh-hannover.de (F.S.); pich.andreas@mh-hannover.de (A.P.); 3 Core Facility Proteomics, Hannover Medical School, Carl-Neuberg-Str. 1, 30625 Hannover, Germany; 4 Department for Rheumatology and Immunology, Hannover Medical School, Carl-Neuberg-Str. 1, 30625 Hannover, Germany; behrens.georg@mh-hannover.de; 5 German Center for Infection Research (DZIF), Partner Site Hannover-Braunschweig, Inhoffenstr. 7, 38124 Braunschweig, Germany; 6 Heart and Diabetes Center North Rhine-Westphalia, Institute for Laboratory and Transfusion Medicine, Ruhr University Bochum, Georgstr. 11, 32545 Bad Oeynhausen, Germany; jkuhn@hdz-nrw.de

**Keywords:** adverse drug reaction, HLA-B*57:01, abacavir, proteome, hypersensitivity, T cell receptor

## Abstract

Type B adverse drug reactions (ADRs) represent a significant threat as their occurrence arises unpredictable and despite proper application of the drug. The severe immune reaction Abacavir Hypersensitivity Syndrome (AHS) that arises in HIV^+^ patients treated with the antiretroviral drug Abacavir (ABC) strongly correlates to the presence of the human leukocyte antigen (HLA) genotype HLA-B*57:01 and discriminates HLA-B*57:01^+^ HIV^+^ patients from ABC treatment. However, not all HLA-B*57:01^+^ HIV^+^ patients are affected by AHS, implying the involvement of further patient-specific factors in the development of AHS. The establishment of a reliable assay to classify HLA-B*57:01 carriers as ABC sensitive or ABC tolerant allowed to investigate the T cell receptor (TCR) Vβ chain repertoire of effector cells and revealed Vβ6 and Vβ24 as potential public TCRs in ABC sensitive HLA-B*57:01 carriers. Furthermore, distinct effects of ABC on the cellular proteome of ABC sensitive and tolerant volunteers were observed and suggest enhanced activation and maturation of dentritic cells (DC) in ABC sensitive volunteers. Analysis of ABC-naïve cellular proteomes identified the T cell immune regulator 1 (TCIRG1) as a potential prognostic biomarker for ABC susceptibility and the involvement of significantly upregulated proteins, particularly in peptide processing, antigen presentation, interferon (IFN), and cytokine regulation.

## 1. Introduction

Adverse drug reactions (ADR) are harmful but unintended reactions triggered by the application of a drug [1]. ADRs are divided into augmented “on-target” type A and idiosyncratic “off-target” type B reactions [2,3]. While type A reactions are dose-dependent, based on pharmacological effects of drugs, and rarely life-threatening [4,5], type B reactions arise despite proper application of the drug, occur especially in patients with a certain predisposition, and result in high mortalities [6,7]. Type B ADRs are immunologically mediated and include immediate onset antibody-mediated reactions and delayed hypersensitivity reactions (DHRs) [8]. DHRs are often caused by T cell-mediated immune activation and typically manifest within hours or weeks post drug administration, with cutaneous symptoms affecting the skin, lung, heart, liver, or kidney. The clinical appearance ranges from nonlethal maculopapular exanthemas (MPE) to life-threatening systemic syndromes, such as toxic epidermal necrolysis (TEN) or Stevens-Johnsen-Syndrome (SJS) [8,9,10]. In recent years, it became apparent that DHRs are associated with distinct alleles of the highly polymorphic human leukocyte antigen (HLA) system [11,12,13,14].

HLA molecules are highly polymorphic cell surface glycoproteins representing a fundamental part of the immune system. They are involved in immune responses through presentation of self- or pathogen-derived peptides to T lymphocytes. Based on the origin of the presented peptide, the HLA system provides information of the individual health condition to prevent and combat infections. The peptide binding region (PBR) of HLA is the region with the highest variability and a basis for the diverse presented immunopeptidome [15,16]. Almost all nucleated cells express HLA class I molecules, which are presented to CD8^+^ T cells. With their respective T cell receptor (TCR), CD8^+^ T cells recognize the trimeric complex of HLA class I heavy chain, light chain, and endogenously processed peptide and discriminate between self- and pathogen-derived or foreign peptides [17,18]. 

Drugs are usually much smaller than peptides, which are bound to HLA molecules; hence, they are not able to induce immune responses. However, to understand how small drug molecules may activate the immune system in an HLA-dependent manner, three models have been postulated. According to the hapten/prohapten model, the drug or its reactive metabolite covalently binds to a host protein or peptide and generates an immunogenic, haptenated, de novo peptide that is presented on HLA molecules. Peptide-independent, non-covalent binding of the drug to either the TCR or HLA protein that directly activates T cell responses is referred to as the pharmacological interaction with the immune receptors (p-i) model. The third model describes an altered peptide repertoire caused by altered biochemical features of the PBR through drug binding (altered repertoire model), resulting in the presentation of neo-antigens on HLA molecules [2,3,8]. 

Unintended activation of immune effector cells post drug administration in an HLA-allele-dependent manner is impossible to preclude post approval of a drug. The variability of the HLA system makes the implication of HLA genotypes in clinical trials prior to drug approval unfeasible. Hence, dissolving the task of personalized drug safety in the context of HLA/drug and CD8^+^ T cell engagement is like searching for a needle in a haystack. The HLA-B*57:01-mediated ADR post Abacavir medication represents a strong paradigm for the analysis of drug-induced intracellular conditions. The nucleoside analogue reverse transcriptase inhibitor Abacavir (ABC) used for the treatment of HIV-1 infections causes severe hypersensitivity reactions in certain patients. ABC hypersensitivity syndrome (AHS), which is strongly correlated to the carriage of HLA-B*57:01, was the first discovered HLA-mediated DHR [19,20,21]. Screening for HLA-B*57:01 prior to treatment with ABC is highly recommended by the Food and Drug Administration (FDA), and ABC medication is contraindicated for HLA-B*57:01^+^ HIV-1^+^ patients [22].

The biophysical mechanism of AHS has already been elucidated. The specific binding of ABC within the peptide binding region of HLA-B*57:01 molecules alters the biochemical features of the F pocket. As a result, self-peptides that have not been involved in thymic selection bind to HLA-B*57:01 and are presented to autologous CD8^+^ T cells [23,24,25]. Due to missing T cell tolerance to these novel self-peptides, autologous CD8^+^ T cells elicit polyclonal T cell responses against the neo-antigens [26], resulting in a graft-versus-host disease-like reactions.

Typing for HLA-B*57:01 prevented the occurrence of immunologically confirmed AHS, with a negative predictive value (NPV) of 100%. However, the positive predictive value (PPV) of 49.7% implies ABC sensitive or ABC tolerant characteristics of HLA-B*57:01^+^ HIV-1^+^ patients [27,28], suggesting that further, patient-specific factors may contribute to the development of AHS. Dissolving and appreciating these patient-specific factors is the challenge depicted in this study. Since ABC always occupies the peptide binding groove of each available HLA-B*57:01 molecule, resulting in alteration of the recruited self-peptide repertoire, the availability of ABC tolerant HLA-B*57:01 carriers seems inapprehensible.

Initial evidence was provided by examining HLA-B*57:01-linked ABC sensitivity and tolerance in vivo in transgenic (tg) mice. While immunocompetent tg mice tolerated ABC treatment, depletion of CD4^+^ T lymphocytes, resulting in greater maturation of dendritic cells (DCs) and enhanced antigen-presenting cell (APC) co-stimulation, favored AHS in the respective mice [29,30]. Furthermore, a retrospective analysis of ABC sensitive and ABC tolerant HLA-B*57:01^+^ HIV-1^+^ patients defined a soluble cluster of differentiation 14 (sCD14) and endoplasmic reticulum aminopeptidase 1 (ERAP1) allotypes as new potential genetic predictors for ABC tolerance. ABC sensitivity significantly correlates with a functional polymorphism within the *CD14* gene, resulting in an increased amount of sCD14. Moreover, HLA-B*57:01^+^ HIV-1^+^ patients displayed distinct functional haplotypes of ERAP1 whereby ABC tolerant patients exhibit hypoactive trimming ERAP1 allotypes that conceivably influence the immunopeptidome of the respective patient through differential antigen processing [31,32].

The HLA/drug specific TCR is an essential component for HLA-mediated DHRs and is also a potential risk factor [8]. Due to the high diversity of the TCR repertoire, most TCRs involved in T cell responses exhibit a unique or private specificity in one individual. Nevertheless, in some T cell responses, shared or public, TCRs are observed in multiple individuals [33]. A drug-specific public αβTCR was already observed for HLA-B*15:02-associated Carbamazepine (CBZ)-induced SJS/TEN. Interestingly, this TCR was only present in CBZ sensitive patients with the genotype HLA-B*15:02 [34].

The present study aims to provide a step towards a comprehensive understanding of differential immunological AHS through immune profiling of ABC susceptible patients with the HLA-B*57:01 genotype. For this purpose, we established a reliable assay for the classification of ABC sensitive and ABC tolerant HLA-B*57:01^+^ healthy volunteers. Based on this classification, we investigated the Vβ TCR repertoire of the respective volunteers to identify a potential public ABC-specific Vβ TCR among ABC sensitive volunteers. Furthermore, we performed full proteome analysis using mass spectrometry to compare the effect of ABC treatment on the proteome of ABC sensitive and ABC tolerant volunteers in order to describe further prognostic risk factors that might contribute to the development of AHS. Knowledge about the involvement of genetic and cellular risk factors in HLA-mediated DHRs will help to improve drug safety and facilitate personalized treatment of patients.

## 2. Materials and Methods

### 2.1. Maintenance of Cell Lines

The human B-lymphoblastoid cell line *LCL721.221* (LGC Promochem, Wesel, Germany; HLA class I^−^/TPN^+^) and peripheral blood mononuclear cells (PBMCs) from healthy volunteers were maintained in RPMI 1640 (Lonza, Basel, Switzerland), supplemented with 10% heat-inactivated fetal calf serum (FCS, Lonza), 2 mM L-glutamine (c. c. pro, Oberdorla, Germany), 100 U/mL penicillin, and 100 µg/mL streptomycin (c. c. pro) at 37 °C and 5% CO_2_.

### 2.2. Cloning of Constructs Encoding for HLA-B*57:01

Constructs encoding for full length HLA-B*57:01 (mHLA-B*57:01, Exon 1–7) were generated from cDNA of an HLA-B*57:01^+^ donor via PCR, as previously described [35]. The cDNA encoding for mHLA-B*57:01 was cloned into the lentiviral vector pRRL.PPT.SFFV.mcs.pre and checked through sequencing.

### 2.3. Stable Transduction of LCL721.221 Cells with Lentivirus Encoding for HLA-B*57:01

*HEK293T* cells were transfected with the HLA-B*57:01 encoding transfer vector, the packaging and envelope vectors psPAX, and pmD2G. A produced virus was used to stably transduce *LCL721.221* cells, as previously described [36,37]. Expression of HLA-B*57:01 on the cell surface of *LCL721.221* cells was confirmed by flow cytometry using the mab anti-human HLA-I w6/32 (eBioscience Inc., San Diego, CA, USA).

### 2.4. CFSE-Based Cytotoxicity Assay (CTA)

LCL721.221/mHLA-B*57:01 were incubated with or without 50 µg/mL ABC for 24 h. Afterwards, cells were washed with phosphate-buffered saline (PBS, Lonza). ABC-preincubated target cells were labelled with 0.2 µM CFSE (5(6)-CFDA, SE, Life Technologies^TM^, Carlsbad, CA, USA) and untreated target cells with 2 µM CFSE for 15 min at 37 °C in 5% CO_2_. Cells were pelleted at 1500 rpm for 5 min and resuspended in RPMI 1640 medium, supplemented with 10% human serum type AB (PAN-Biotech GmbH, Aidenbach, Germany). The two target cell populations were seeded 1:1 into a 48-well microtiter plate. 

Peripheral blood of HLA-B*57:01^+^ donors (Table 1) was obtained after informed consent, as approved by the Ethics Committee of Hannover Medical School. LymphoSep^TM^ (c. c. pro) density separation was performed in order to isolatePBMCs. CD8^+^ cells were isolated by negative selection using the human CD8^+^ T Cell Isolation Kit (Miltenyi Biotec GmbH, Bergisch Gladbach, Germany) and added to the target cells at a 10:1 effector-target ratio. The plate was incubated at 37 °C and 5% CO_2_. After 4 h, cell viability of the ABC-treated and untreated target cells was detected by 7-AAD (Life Technologies^TM^) staining via flow cytometry. ABC-specific lysis was calculated as follows:

% specific cell death = [100 × % dead target cells − % spontaneous dead targets]/100 − % spontaneous dead targets(1)

### 2.5. Analysis of T Cell Receptor (TCR) Vβ-Chain Repertoire

PBMCs were isolated from the peripheral blood of four classified ABC sensitive and ABC tolerant HLA-B*57:01^+^ healthy blood donors, as described previously. CD8^+^ cells were isolated directly after LymphoSep^TM^ density separation to obtain naïve CD8^+^ cells. Additionally, PBMCs were incubated in RPMI 1640, supplemented with 10% human serum type AB for 48 h in the absence (to obtain unspecific CD8^+^ cells) or presence of 50 µg/mL ABC (to obtain ABC-specific CD8^+^ cells) prior to isolation of CD8^+^ cells. In order to isolate total RNA, 5 × 10^6^ CD8^+^ cells were lysed in RLT buffer (Qiagen N. V., Venlo, The Netherlands) with β-mercaptoethanol (Sigma Aldrich^®^ Co. LLC, St. Louis, MS, USA) and RNA isolation was performed according to *QIAquick* RNeasy Mini Kit (Qiagen N. V.) manufacturer’s manual. A total of 1.5 µg RNA was applied for cDNA synthesis according to High-Capacity cDNA Reverse Transcription Kit (Applied Biosystems by Life Technologies, Carlsbad, CA, USA) manufacturer’s manual. A quantitative RT-PCR (qRT-PCR) was performed, as described previously [38]. For each reaction, 15 ng cDNA was used, and the qRT-PCR was performed in triplicate.

### 2.6. Mass Spectrometric Detection of ABC in Solution

For ABC tracking in cell lysates, 5 × 10^5^ *LCL721.221* cells were incubated with or without 50 µg/mL ABC for 0 h, 24 h, 48 h, and 72 h. Cells were lysed in 250 µL RIPA buffer, as described by Ho et al. [39]. Sample preparation and mass spectrometric detection of ABC was conducted as described previously [40].

### 2.7. Mass Spectrometric Analysis of ABC-Induced Modifications in PBMCs of ABC Sensitive and ABC Tolerant HLA-B*57:01 Carriers

For proteome analysis, 1 × 10^7^ PBMCs of three classified ABC sensitive and three ABC tolerant HLA-B*57:01^+^ healthy blood donors were treated with 100 µg/mL ABC for 48 h. After 24 h, drug treatment was repeated. Cells were harvested and lysed in RIPA buffer, as previously described [37]. Protein concentration was calculated by using the Bicinchoninic acid assay (BCA) Protein Quantitation Kit (Interchim, San Diego, CA, USA). Sample preparation and MS analysis was performed using a highly resolving nanoLC system and Orbitrap MS, as previously described by Simper et al. [41]. 

## 3. Results

### 3.1. Classification of HLA-B*57:01^+^ Donors as ABC Sensitive or ABC Tolerant

**Figure 1:**
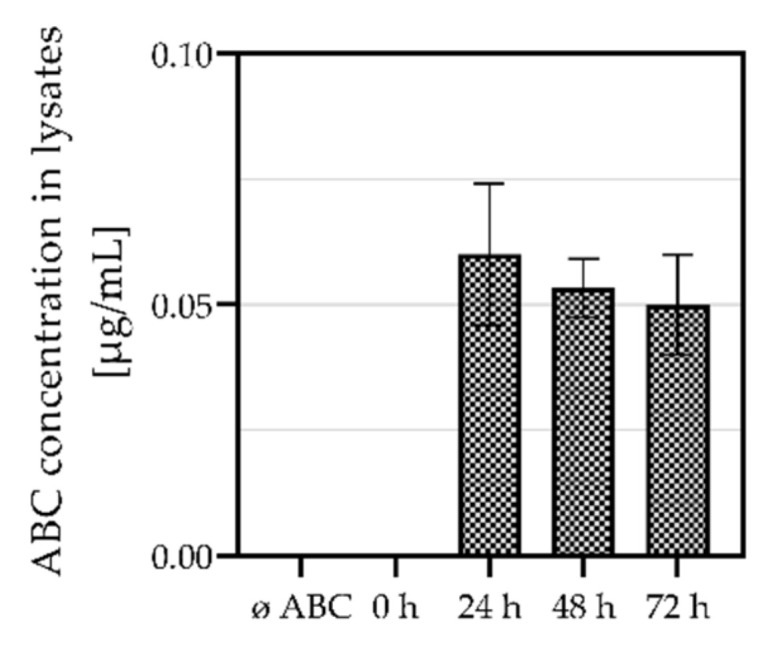
ABC concentrations in cell lysates following various incubation periods. *LCL721.221* cells were cultured in the absence or presence or absence (ø ABC) of 50 µg/mL ABC in three technically independent replicates (*n* = 3) and ABC amounts were measured by UPLC-MS/MS.

To classify HLA-B*57:01^+^ healthy volunteers into ABC sensitive or ABC tolerant, an allogenic in vitro CTA was performed. LCL721.221/mHLA-B*57:01 cells were incubated in the absence or presence of ABC and used as target cells. To verify ABC uptake in *LCL721.221* cells and to determine the incubation time sufficient for absorption of ABC, ABC concentration in *LCL721.221* cell lysates was detected after various incubation periods (Figure 1). The highest ABC amount was detected after 24 h of incubation (0.06 µg/mL). Longer incubation times led to a decrease in detected ABC amounts, thus we used 24 h of incubation for ABC pre-incubation of target cells. 

Distinction of ABC-treated and untreated target cells was achieved by differential CFSE staining. CD8^+^ effector cells isolated from PBMCs of HLA-B*57:01^+^ volunteers were added in an effector-target ratio of 10:1 and incubated for 4 h. Afterwards, target cell viability was determined (Figure S1) and the cytotoxic potential of CD8^+^ cells towards untreated and ABC-treated target cells was calculated. As a positive control for all assays, an HIV^+^ HLA-B*57:01^+^ patient, who developed a credibly documented AHS in 2007, was used. CD8^+^ cells of this patient exhibited increased cytotoxicity towards ABC-treated target cells (0.7% difference in cytotoxic potential of CD8^+^ cells against ABC-treated compared to untreated target cells, Figure S1C). Furthermore, two ABC-naïve HIV^+^ HLA-B*57:01^+^ patients were analyzed for their T cell reactivity post ABC exposure. Patient A was classified as ABC sensitive, whereas patient B showed no reactivity towards ABC (Figure S2).

**Figure 2:**
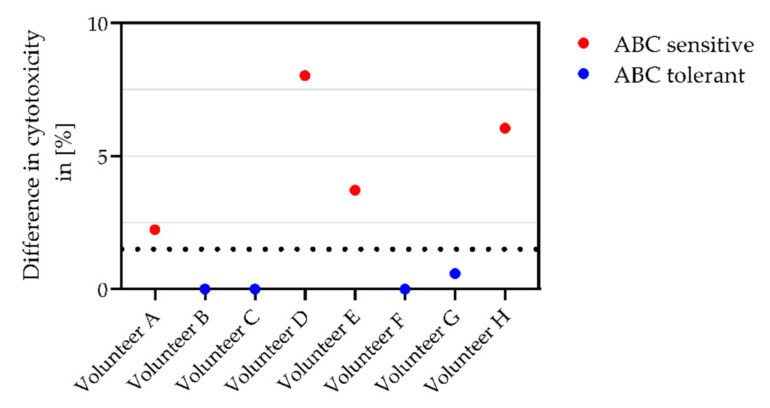
Classification of HLA-B*57:01^+^ healthy volunteers as ABC sensitive (red) or ABC tolerant (blue). Depicted is the cytotoxic potential (% of dead cells) of CD8^+^ cells following ABC treatment of target cells. CTA was conducted in two biologically and technically independent replicates (*n* = 4). Dash line shows the threshold for classification.

The ABC-specific cytotoxic potential of CD8^+^ cells was calculated; HLA-B*57:01^+^ healthy volunteers were classified as ABC sensitive or ABC tolerant (Figure 2). The threshold for ABC sensitivity was set to 0.7% difference in cytotoxicity, according to the negative control. 

### 3.2. TCR Vβ-Chain 6 and 24 Are Potential Candidates for Public ABC-Specific TCRs

To identify phenotypical differences in CD8^+^ cells causing ABC sensitivity or tolerance in HLA-B*57:01^+^ carriers, PBMCs of four classified ABC sensitive volunteers and four ABC tolerant volunteers were stimulated in the presence or absence of ABC for 48 h. Phenotypical alterations of 24 TCR Vβ chains in CD8^+^ cells were analyzed via RT-qPCR. The % TCR Vβ usage in ABC-specific and non-specific CD8^+^ cells was normalized to % TCR Vβ usage in autologous naïve CD8^+^ cells. Differences in % TCR Vβ usage of ABC-specific to non-specific CD8^+^ cells (ΔABC-specific/non-specific) were compared in ABC sensitive and ABC tolerant HLA-B*57:01 carriers.

**Figure 3:**
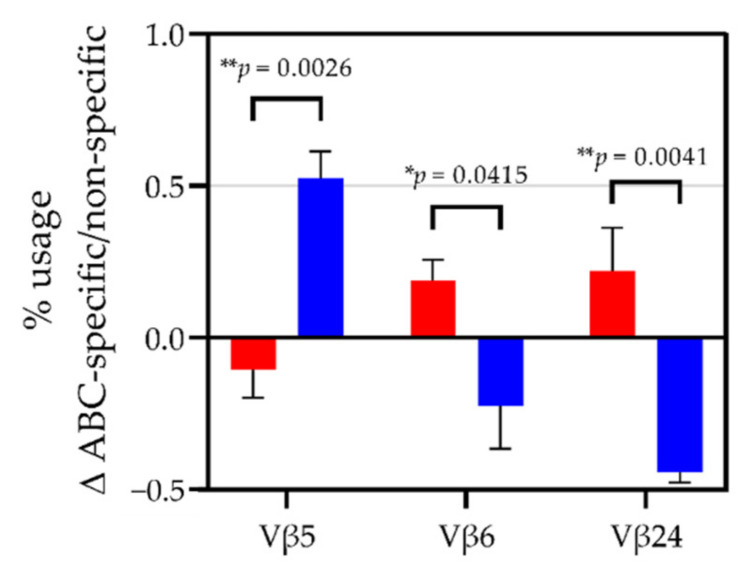
Difference in relative TCR Vβ5, Vβ6, and Vβ24 mRNA expression in ABC-specific CD8^+^ cells compared to non-specific CD8^+^ cells of ABC sensitive and ABC tolerant HLA-B*57:01^+^ healthy volunteers. TCR Vβ repertoires of CD8^+^ cells of classified ABC sensitive and ABC tolerant healthy HLA-B*57:01 carriers were analyzed before and after 48 h of ABC-stimulation or unspecific stimulation. The relative Vβ mRNA expression was normalized to the constant region of the TCR and the difference in transcription in ABC-specific to non-specific CD8^+^ cells after 48 h was normalized to Vβ mRNA expression of naïve CD8^+^ cells. Bars and error bars represent mean ± SEM. Experiments were performed in four biologically and three technically independent replicates (*n* = 12). * *p* ≤ 0.05, ** *p* ≤ 0.01.

In ABC sensitive HLA-B*57:01 carriers, TCR Vβ5 usage in ABC-specific cells compared to non-specific CD8^+^ cells decreased, whereas in ABC tolerant HLA-B*57:01 carriers, Vβ5 usage increased. In contrast, ABC-specific CD8^+^ cells displayed enhanced TCR Vβ6 and Vβ24 expression in ABC sensitive HLA-B*57:01 carriers and decreased usage in ABC tolerant HLA-B*57:01 carriers (Figure 3), suggesting the TCR Vβ chains as potential candidates for public TCRs in response to ABC.

### 3.3. Impact of ABC Treatment on the Proteome of PBMCs Significantly Differs in ABC Sensitive and ABC Tolerant HLA-B*57:01 Carriers

**Figure 4:**
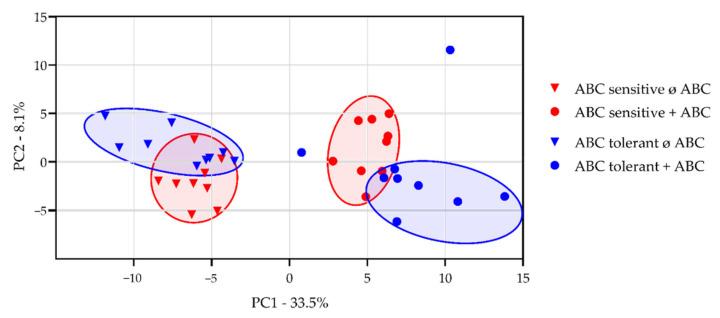
Principal component analysis (PCA) of proteins that were significantly altered (*p* < 0.05) in PBMCs of each three classified ABC sensitive (red) and ABC tolerant (blue) HLA-B*57:01^+^ healthy donors following ABC treatment. PBMCs were incubated without (ø) or with (+) ABC for 48 h in three biologically and three technically independent replicates (*n* = 9).

To investigate the impact of ABC on the cellular proteome and identify potential differences between ABC sensitive and ABC tolerant HLA-B*57:01^+^ volunteers, an LC-MS-based approach was chosen. PBMCs of classified volunteers were cultured in the absence or presence of ABC for 48 h. ABC uptake of PBMCs was verified by UPLC-MS/MS and corresponded to 2 µg/mL (≙28 µM). MaxQuant software [42], and label-free quantification (LFQ) was applied to determine relative protein abundance. Since each HLA-B*57:01^+^ volunteer exhibited a distinct proteome with up- and downregulation of specific proteins, protein abundance was normalized on the median proteome intensity of each volunteer. A principal component analysis (PCA) was performed to visualize significant differences between no drug treatment and ABC treatment, as well as between ABC sensitive and ABC tolerant HLA-B*57:01 carriers. The impact of ABC-treatment compared to no treatment was clearly distinguishable in HLA-B*57:01^+^ ABC sensitive and ABC tolerant volunteers (Figure 4). 

### 3.4. Proteins Upregulated in PBMCs from ABC Sensitive Compared to ABC Tolerant Healthy HLA-B*57:01 Carries following ABC Treatment Are Involved Various Immune Responses

To identify differences in the cellular responses of classified ABC sensitive and ABC tolerant HLA-B*57:01^+^ volunteers to ABC treatment, the proteomic content following ABC treatment was normalized to the proteomic content of untreated PBMCs and normalized protein amounts were compared in ABC sensitive to ABC tolerant volunteers. 

**Figure 5:**
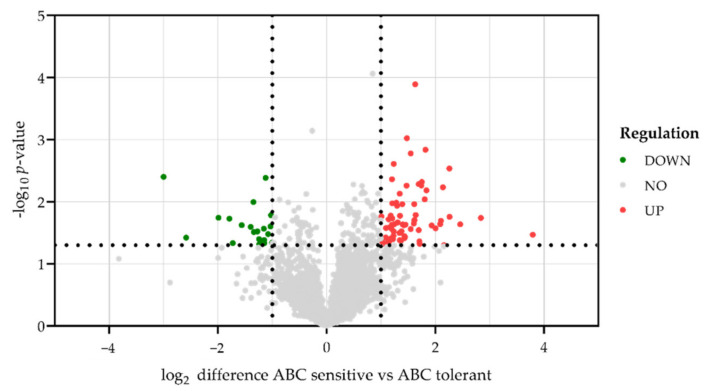
Differences in protein abundance in PBMCs of each three classified ABC sensitive and ABC tolerant HLA-B*57:01^+^ healthy donors following ABC treatment. The volcano plot illustrates significantly differentially abundant proteins after ABC treatment of three biologically and three technically independent replicates (*n* = 9). The log_2_ fold change of ABC sensitive compared to ABC tolerant HLA-B*57:01^+^ volunteers is plotted against the −log_10_ *p*-value. Proteins were regarded as regulated in ABC sensitive donors from factor ± 1.0 and *p* < 0.05. Downregulated proteins are labelled in green, unregulated proteins are colored in grey, and upregulated proteins are given in red.

MS-based proteomic analysis revealed 4297 protein groups; 2561 of them could be quantified in all replicates. Exclusively significant regulated proteins (*p*-value < 0.05) and proteins that were at least altered by factor log_2_ ± 1.0 in ABC sensitive compared to ABC tolerant HLA-B*57:01^+^ volunteers were regarded as regulated. In total, 67 quantified proteins were significantly upregulated, and 22 quantified proteins were significantly downregulated in PBMCs of ABC sensitive carrier compared to ABC tolerant HLA-B*57:01 carriers (Figure 5). The strongest upregulated protein was the 60S acidic ribosomal protein P1 (RPLP1), followed by the serine/threonine-protein phosphatase 2A (PPP2R2C) and the beta 1 chain of the HLA class I molecule HLA-DQ (HLA-DQB1). Furthermore, the tyrosine-protein kinase Lyn (LYN), macrosialin (CD68) and the E3 ubiquitin-protein ligase ZFP91 were among the 10 strongest upregulated proteins (Table 2).

The software Ingenuity Pathway Analysis (IPA, Version 70750371, QIAGEN, Redwood City, CA, USA) was utilized to identify upstream regulators that explain the observed proteome changes. Thereby, the tyrosine-protein kinase ZAP70 and The TCR ζ-chain (CD247) were identified as upstream regulators that trigger the upregulation of several proteins in ABC sensitive compared to ABC tolerant HLA-B*57:01^+^ volunteers. 

### 3.5. Proteomic Content of Untreated PBMCs from ABC Sensitive and ABC Tolerant Healthy HLA-B*57:01 Carriers Varies Significantly

**Figure 6:**
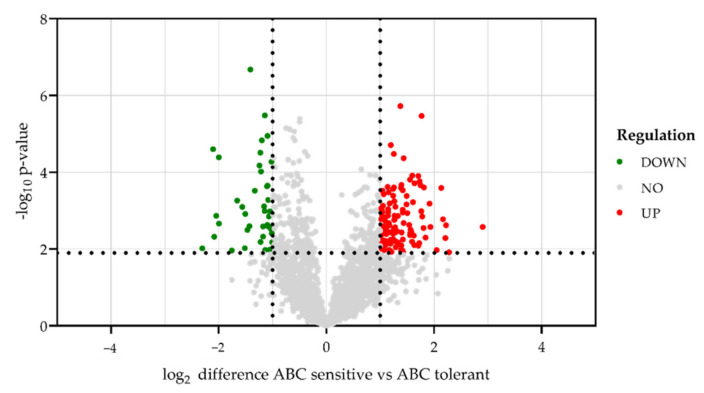
Differences in protein abundance in untreated PBMCs of each three classified ABC sensitive and ABC tolerant HLA-B*57:01^+^ healthy donors. The volcano plot illustrates significantly differentially abundant proteins of three biologically and three technically independent replicates (*n* = 9). The log_2_ fold change of ABC sensitive compared to ABC tolerant HLA-B*57:01^+^ donors is plotted against the −log_10_ *p*-value. Proteins were regarded as regulated in ABC sensitive volunteers from factor ±1.0 and *p* < 0.05. Downregulated proteins are labelled in green, unregulated proteins are colored in grey, and upregulated proteins are given in red.

PCA demonstrated that the proteome profile of untreated PBMCs from ABC sensitive and ABC tolerant HLA-B*57:01^+^ healthy volunteers is clearly distinguishable (Figure 4), suggesting a potential biomarker among the differentially regulated proteins of untreated PBMCs. Comparison of the LC-MS-based proteomic analysis of untreated PBMCs from ABC sensitive and ABC tolerant healthy HLA-B*57:01 carriers revealed 4297 protein groups, and 2619 of them could be quantified in all replicates. Significantly regulated proteins were determined by a permutation-based false discovery rate (FDR), and proteins at least altered by factor log_2_ ± 1.0 in ABC-responders compared to ABC-non-responders were regarded as regulated. In total, 120 quantified proteins were significantly upregulated, and 41 quantified proteins were significantly downregulated in PBMCs from ABC sensitive compared to ABC tolerant HLA-B*57:01 carriers (Figure 6). The strongest significantly upregulated proteins in ABC sensitive volunteers were the basement protein nidogen-1 (NID1) and the phospholipase B domain containing 1 (PLBD1). The T cell immune regulator 1 (TCIRG1), which also belongs to the 10 strongest upregulated proteins in ABC sensitive compared to ABC tolerant HLA-B*57:01 carriers, is a subunit of the vacuolar ATPase (V-ATPase) complex (Table 3).

**Figure 7:**
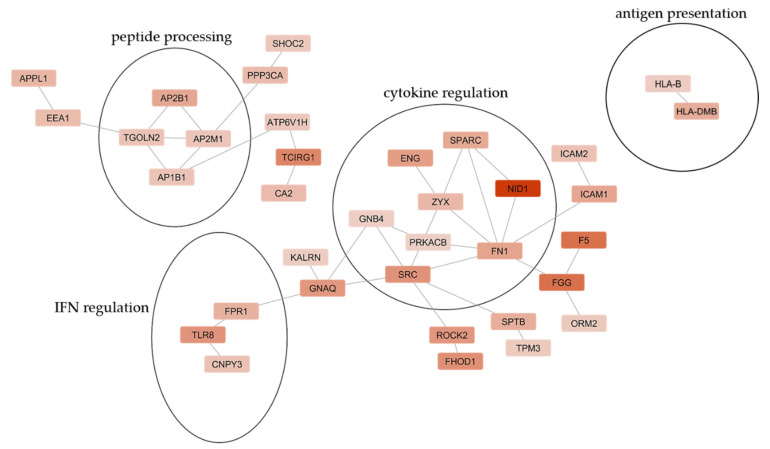
Protein-protein interaction network of significantly up- and downregulated proteins in ABC sensitive compared to ABC tolerant HLA-B*57:01 carriers and cellular processes they are involved in (based on GO/KEGG). The network was constructed by the STRING Database (Version 11.5) and visualized using Cytoscape (Version 3.8.2). Significant regulated proteins determined by permutation-based FDR and at least altered by factor log_2_ + 1 were considered. Upregulated proteins are illustrated in red. Color intensity reflects the log_2_ fold difference between ABC sensitive compared to ABC tolerant HLA-B*57:01 carriers.

We created a protein-protein interaction (PPI) network via the STRING Database that illustrates clusters of significantly upregulated proteins in ABC sensitive compared to ABC tolerant HLA-B*57:01^+^ healthy volunteers that are particularly involved in peptide processing, antigen presentation, cytokine, and IFN regulation (Figure 7).

## 4. Discussion

Type B ADRs display a major economic burden for the public health care system as their occurrence arises despite proper application of the drug and is unpredictable. Thus, ADRs lead to life-threatening consequences for patients, as well as enormous cost for the pharmaceutical industry due to hospital admissions and inpatient treatment [6]. Discovering the contribution of HLA molecules to DHRs was an important milestone of research; however, low PPVs based on case control studies of several HLA-associated DHRs [32] indicate the contribution of further patient-specific risk factors. Thus, fundamental comprehension of the molecular mechanisms is inevitable and will increase drug safety. Due to the exceptional specificity of AHS and HLA-B*57:01, ABC is a great candidate to comprehensively exploit the mechanism of differential immunological DHRs. 

A Tg mouse model for HLA-B*57:01-linked ABC drug tolerance and reactivity by Cardone et al. in 2018 revealed the necessity of enhanced DC maturation and APC co-stimulation through the depletion of CD4^+^ cells to provoke ABC-reactive CD8^+^ T cells with the potential to cause AHS in the respective mice [29]. In addition, increased sCD14 levels due to a SNP in the respective gene and more efficient ERAP1 allotypes were defined as new potential risk factors for the development of AHS [31,32], suggesting a contribution of activated monocytes and enhanced antigen processing to ABC sensitivity. sCD14 is released by monocytes upon activation [43] and described as a predictive marker for mortality and morbidity during HIV infection [44], whereas ERAP1 is involved in the N-terminal trimming of peptides in the endoplasmic reticulum, which are presented by HLA [45]. 

In the present study, we established a standardized protocol for patient screenings by an allogenic in vitro CTA, utilizing LCL721.221/mHLA-B*57:01 cells as target cells that ensured constant HLA-B*57:01 expression on the cell surface and allowed the classification of HLA-B*57:01 healthy volunteers as ABC sensitive or ABC tolerant. Chessman et al. demonstrated in 2008 that ABC also induces the activation and proliferation of CD8^+^ T cells from ABC-naïve healthy HLA-B*57:01^+^ blood donors in vitro [26]. For validation, we performed our assay with an HIV^+^ HLA-B*57:01^+^ patient who previously developed AHS as a positive control and, although HLA-B*57:03 is a closely related natural allotype of HLA-B*57:01, an HIV^+^ HLA-B*57:03^+^ patient as a negative control. Application of this assay enabled the identification of four volunteers exhibiting ABC sensitivity and four volunteers exhibiting ABC tolerance. 

Based on this classification, we investigated and compared ABC sensitive and ABC tolerant HLA-B*57:01^+^ volunteers for their TCR Vβ-chain repertoire via RT-qPCR with 24 TCR Vβ-specific primer pairs. For ABC-specific T cells, it has been observed that polyclonal TCR usage is consistent with the altered repertoire model [23]. However, we aimed to identify potential phenotypic differences of CD8^+^ effector cells that might clarify why some, but not all HLA-B*57:01, carriers develop ABC-induced hypersensitivity reactions. TCR bias has been observed in many antigen-specific responses and in autoimmunity and alloreactivity. We proposed differential type 1 TCR bias in ABC sensitive and tolerant HLA-B*57:01 carriers. This bias is characterized by the selection of single TCR Vβ-chains with little or no conservation in CDR3 regions and might originate from thymic selection, immune response initiation, or persistent infection [46]. Differential type 1 TCR bias would correspond with the altered repertoire model described for AHS. Among ABC sensitive volunteers, we identified TCR Vβ6 and Vβ24 as potential candidates for public ABC-specific TCRs that elicit ABC-induced immune responses in multiple unrelated individuals and might contribute to ABC-mediated immunoreactivity against autologous cells. Recently, Pan et al. discovered a public TCR for CBZ-mediated SJS/TEN in HLA-B*15:02^+^ patients [34]. 

LC-MS-based proteome analysis of untreated and ABC-treated PBMCs from ABC sensitive and tolerant HLA-B*57:01 carriers demonstrated significant differences. We also performed two separate PCAs to show that the proteomic content of ABC-naïve and ABC-treated PBMCs of ABC sensitive and tolerant volunteers are clearly distinguishable (Figure S3). ABC uptake was ensured by HPLC-MS/MS prior to proteome analysis, and the amount of ABC in cell lysates (2 µg/mL) was consistent with the physiological concentration of ABC within the human body (peak concentration between 0.88–3.19 µg/mL) [47]. In our study, we particularly focused on upregulated proteins, since protein abundance is closely correlated to peptide presentation [48], thus crucial for influencing T cell activation and subsequently triggering inflammation. Comparison of the effect of ABC treatment on the cellular proteome of ABC sensitive and ABC tolerant HLA-B*57:01^+^ volunteers revealed the strongest upregulation of the ribosomal protein RPLP1 and the serine/threonine-protein phosphatase 2A that catalyzes the removal of phosphate groups from serine and threonine residues. However, we found no correlation between upregulation of these proteins and ABC-mediated hypersensitivity. Strikingly, upregulation of HLA-DQB1 in ABC sensitive HLA-B*57:01 carriers suggested potential involvement of HLA class II in abacavir sensitivity, as initially reported for HLA-DR7 and HLA-DQ3 [19]. We performed HLA class II genotyping of the investigated volunteers for correlation in classification. Since no correlation was found, we did not consider involvement of HLA class II in ABC hypersensitivity. Furthermore, proteins that were involved in a range of immunological processes affecting T cells, monocytes, and DCs were significantly upregulated in cells of ABC sensitive volunteers.

One of these proteins, CD68, is a marker predominantly expressed in monocytes and especially abundant on macrophages [49]. CD68 is preferentially located in late endosomes, suggesting a role in peptide-transport and antigen processing [50]. CD68 is suggested to be a negative regulator for either peptide internalization into late endosomes and loading or HLA class II trafficking by demonstrating that CD68-deficient mononuclear phagocytes display enhanced antigen presentation to CD4^+^ T cells [51,52]. In addition, LYN expression was significantly upregulated. LYN is present in several cell types, predominantly in B lymphocytes and cells of the myeloid linage, and participates in multiple signaling pathways as a positive or negative modulator [53]. In DCs, LYN has been found to positively influence DC maturation and activation. Lyn-deficient DCs displayed a less mature phenotype in response to innate immune stimuli, leading to less IL-12 production and reducing Th1 response priming. Thus, DC maturation seems to be associated with their ability to induce T cell activation [54], with LYN playing a crucial part. 

The atypical E3 ubiquitin-protein ligase ZFP91 ubiquitinates NIK, resulting in the induction of the non-canonical NF-κB pathway. This pathway regulates DC development and maturation and is particularly necessary for DC cross-priming of CD8^+^ T cells. Furthermore, an essential role in the development and maintenance of effector and memory T cells is described for the non-canonical NF-κB pathway [55]. In addition, significantly downregulated proteins were extensively investigated (Table S1), but no obvious protein pattern emerged.

Furthermore, we identified upstream regulators of identified significantly upregulated proteins in ABC sensitive compared to ABC tolerant HLA-B*57:01 carriers via *IPA*. Two upstream regulators, ZAP70 and CD247, are involved in T cell responses [56,57]. CD247 presented by T cells plays a crucial role in adaptive immune responses. ITAMs of CD247 are phosphorylated, following TCR:HLA and CD28:CD80 engagement, and enable recruitment of several molecules. Among them is ZAP70, which is expressed on lymphocytes and essentially involved in the regulation of adaptive immune responses [58]. ZAP70 phosphorylation and activation induces the non-canonical NF-κB pathway [56,57]. 

The proteomic content of untreated PBMCs of ABC sensitive compared to ABC tolerant HLA-B*57:01 carriers exposed significant differences, suggesting the presence of a potential predictive biomarker among the differentially regulated proteins. Analysis of the ABC-naïve proteomes revealed significant upregulation of the key basement membrane protein NID1 that exhibits a role in the organization of skin, muscle, and nervous system basal laminae and is involved in signal transfer through integrins [59]. PLBD1 has been reported to be expressed in neutrophils and monocytes and to generate inflammatory lipid mediators [60], yet the distinct function of this protein is still an enigma. The α3 subunit of the V-ATPase complex TCIRG1 has been identified as 4-fold upregulated in ABC sensitive compared to ABC tolerant HLA-B*57:01 carriers. The V-ATPase complex contributes to various cellular processes, i.e., vesicular trafficking, protein degradation, and endocytosis through acidification of secretory vesicles, lysosomes, and endosomes [61]. TCIRG1 has been reported to be overexpressed in several cancers [62,63] and plays an important role in T cell activation [64]. Mouse experiments revealed an essential function of TCIRG1 in mouse cytotoxic T lymphocytes: acidification through TCIRG1 results in the maturation of cytotoxic granules and their transport to immune synapses [65]. Furthermore, a correlation of TCIRG1 expression and immune infiltration levels has been found in the most malignant brain glioma glioblastoma multiforme [66]. The significant upregulation of TCIRG1 in ABC sensitive compared to ABC tolerant HLA-B*57:01 carriers and the contribution of TCIRG1 in the function of cytotoxic T lymphocytes indicate that TCIRG1 might favor the activation of CD8^+^ T cells in ABC sensitive HLA-B*57:01 carriers. We assume that TCIRG1 might be a prognostic biomarker for ABC-mediated AHS, which requires further investigation. We also investigated the significantly downregulated proteins in untreated PBMCs of ABC sensitive compared to ABC tolerant HLA-B*57:01 carriers, but no clear protein pattern could be detected (Table S2).

We created a PPI network via STRING Database that demonstrated the involvement of significantly upregulated proteins in PBMCs of untreated ABC sensitive compared to ABC tolerant HLA-B*57:01 carriers, especially in peptide processing, antigen presenting, and IFN and cytokine regulation. More efficient peptide processing and antigen presentation correspond to the identification of rather hyperactive ERAP1 allotypes in ABC sensitive HLA-B*57:01^+^ HIV^+^ patients [31,32]. 

In conclusion, the data presented in this study emphasize the complex basis of ABC-mediated AHS with an interplay of several immunological processes involved. Despite the occurrence of the HLA genotype HLA-B*57:01, several patient-specific factors, particularly the activation of DCs, increased antigen presentation, more efficient CD8^+^ T cell cross priming, and enhanced antigen processing contribute to ABC sensitivity. These data will open the door to comprehend the molecular basis of ADRs prior to approval of a medical product and should be implemented in the data of clinical trials.

## Figures and Tables

**Table 1:** HLA class I genotypes of HLA-B*57:01^+^ healthy donors used for all assays.

HLA-B*57:01^+^ Healthy Volunteer	HLA-A Genotype	HLA-B Genotype	HLA-C Genotype	HLA-DPB1 Genotype	HLA-DQB1 Genotype	HLA-DRB1 Genotype
A	A*02:01	B*35:03	C*06:02	04:01	03:02	04:01
	A*11:01	B*57:01	C*12:03	04:02	05:02	16:01
B	A*02:01	B*50:01	C*06:02	04:01	02:01	03:01
		B*57:01			03:03	07:01
C	A*01:01	B*15:01	C*06:02	04:01	02:02	07:01
	A*02:01	B*57:01	C*12:03	04:02	03:03	
D	A*01:01	B*07:02	C*06:02	01:01	07:01	03:03
	A*03:01	B*57:01	C*07:02	04:01	15:01	
E	A*01:01	B*44:03	C*06:02	11:02	02:01	07:01
	A*29:02	B*57:01	C*16:01	23:01		
F	A*01:01	B*44:03	C*06:02	04:02	03:01	07:01
	A*02:01	B*57:01	C*16:01		03:03	11:04
G	A*01:01	B*08:01	C*06:02	01:01	02:01	03:01
		B*57:01	C*07:01	04:01	03:03	07:01
H	A*01:01	B*35:01	C*04:01	04:01	03:03	07:01
	A*11:01	B*57:01	C*06:02	06:01	04:02	08:01

**Table 2:** Strongest upregulated proteins in ABC sensitive compared to ABC tolerant HLA-B*57:01 carriers after ABC treatment.

Protein Name	Gene Code	log_2_ Regulation	*p*-Value
60S acidic ribosomal protein P1	*RPLP1*	3.79	0.034
Serine/threonine-protein phosphatase 2A	*PPP2R2C*	2.84	0.018
HLA class II histocompatibility antigen, DQ beta 1 chain	*HLA-DQB1*	2.46	0.023
Switch-associated protein 70	*SWAP70*	2.26	0.014
Torsin-1A	*TOR1A*	2.26	0.003
Tyrosine-protein kinase Lyn	*LYN*	2.16	0.050
Nurim	*NRM*	2.14	0.006
E3 ubiquitin-protein ligase ZFP91	*ZFP91*	2.10	0.020
Macrosialin	*CD68*	2.09	0.023
Dermcidin	*DCD*	2.00	0.027

**Table 3:** Significantly upregulated proteins in ABC sensitive compared to ABC tolerant HLA-B*57:01 carrier without ABC treatment.

Protein Name	Gene Code	Log_2_ Regulation	*p*-Value
Nidogen-1	*NID1*	2.90	0.003
Phospholipase B-like 1	*PLBD1*	2.28	0.012
Fibrinogen gamma chain	*FGG*	2.22	0.002
Coagulation factor V	*F5*	2.21	0.005
Dysferlin	*DYSF*	2.17	0.002
Proline-serine-threonine phosphatase-interacting protein 2	*PSTPIP2*	2.13	<0.001
Myoferlin	*MYOF*	2.05	0.011
T cell immune regulator 1	*TCIRG1*	1.93	0.003
CAAX prenyl protease 1 homolog	*ZMPSTE24*	1.91	0.001
Plexin-B2	*PLXNB2*	1.84	0.005

## Supplementary Materials

The following are available online at https://www.mdpi.com/article/10.3390/biomedicines10030693/s1, Figure S1: Analysis of the specific cytotoxicity of CD8^+^ cells from HLA-B*57:01 carriers towards ABC-treated and untreated *LCL721.221/HLA-B*57:01* cells. Figure S2: Classification of two HIV^+^ HLA-B*57:01^+^ patients as ABC sensitive (red) or ABC tolerant (blue). Figure S3: Principal component analysis (PCA) of proteins that were significantly altered (*p* < 0.05) in PBMCs of each of the three classified ABC sensitive (red) and ABC tolerant (blue) HLA-B*57:01^+^ healthy donors prior to or following ABC treatment. Table S1: Strongest downregulated proteins in ABC sensitive compared to ABC tolerant HLA-B*57:01 carriers after ABC treatment. Table S2: Strongest downregulated proteins in ABC sensitive compared to ABC tolerant HLA-B*57:01 carriers without ABC treatment.
