# Supplementary material for: Proteomic Profiling and T Cell Receptor Usage of Abacavir Susceptible Subjects
Source: Biomedicines. 2022 Mar 17;10(3):693. doi: 10.3390/biomedicines10030693 (PMC8945713; doi:10.3390/biomedicines10030693)
Supplement: Supplementary file 1 [file biomedicines-10-00693-s001.zip › biomedicines-1574440-supplementary.pdf]

Supplementary Materials

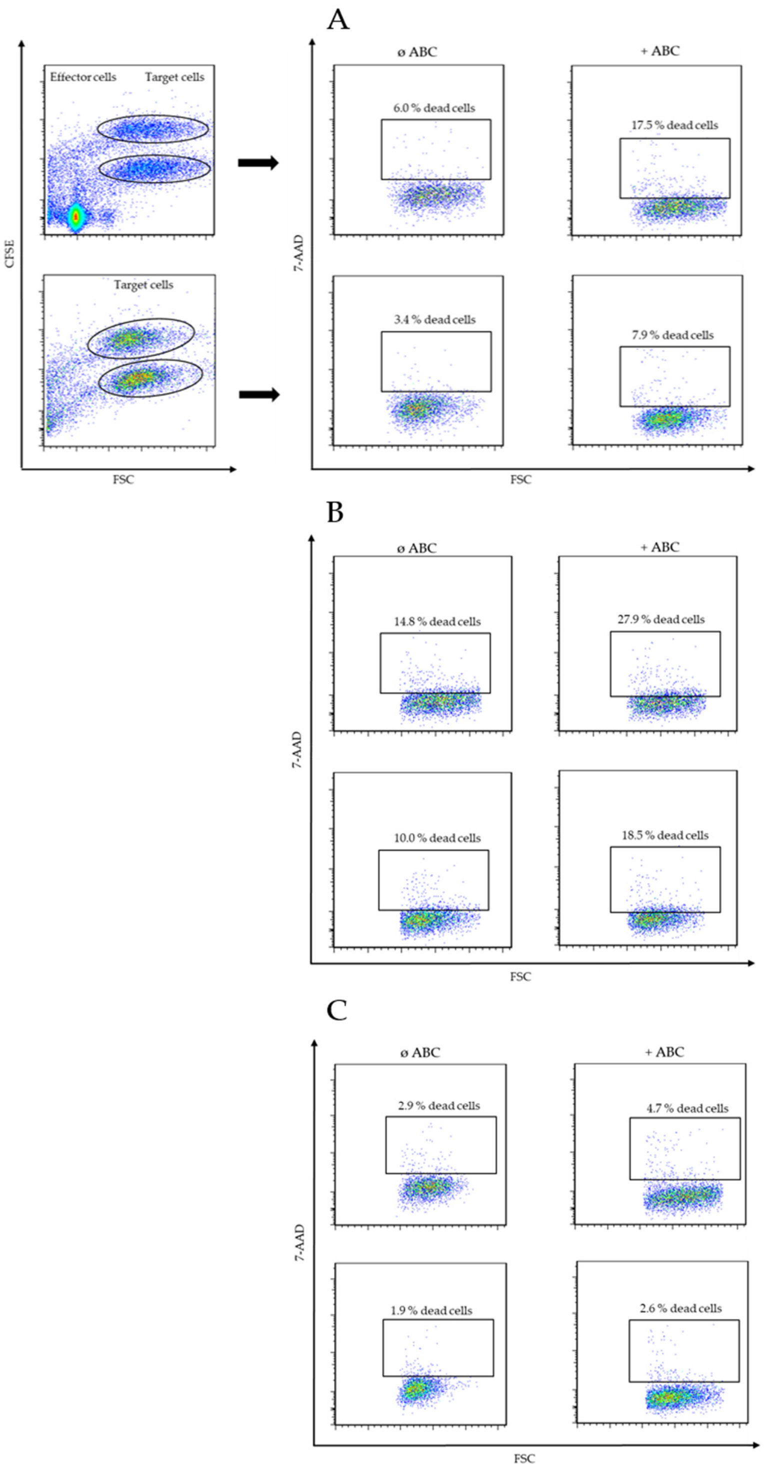

Figure S1: Analysis of the specific cytotoxicity of CD8⁺ cells from HLA-B*57:01 carriers towards ABC-treated and untreated *LCL721.221/HLA-B*57:01* cells. After 4 hours of incubation, target cell viability of target cells that were incubated in the presence or absence of CD8⁺ T cells was determined to calculate the cytotoxic potential of CD8⁺ cells. (**A**) Specific cytotoxicity of CD8⁺ cells of an ABC-sensitive HLA-B*57:01⁺ healthy volunteer. (**B**) Specific cytotoxicity of CD8⁺ cells of an HIV⁺ HLA-B*57:01⁺ patient who developed AHS in 2007 (positive control). (**C**) Specific cytotoxicity of CD8⁺ cells of an HIV⁺ HLA-B*57:03⁺ patient (negative control).

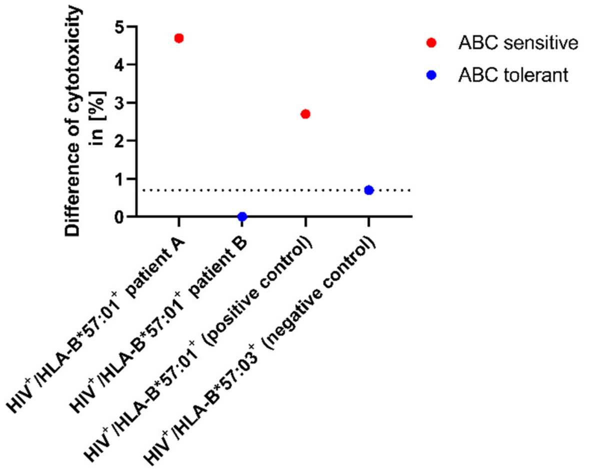

Figure S2: Classification of two HIV+ HLA-B*57:01+ patients as ABC sensitive (red) or ABC tolerant (blue). Depicted is the cytotoxic potential (% of dead cells) of CD8+ cells following ABC treatment of target cells. CTA was conducted in two technically independent replicates (*n* = 2). An HIV+ HLA-B*57:01+ patient who developed a credibly documented AHS in 2007 was used as a positive control that showed 2.7% difference in cytotoxicity whereas an HIV+ HLA-B*57:03+ patient was used as a negative control that showed 0.7% difference in cytotoxicity. Dash line shows the threshold for classification that was set based on the negative control of this assay.

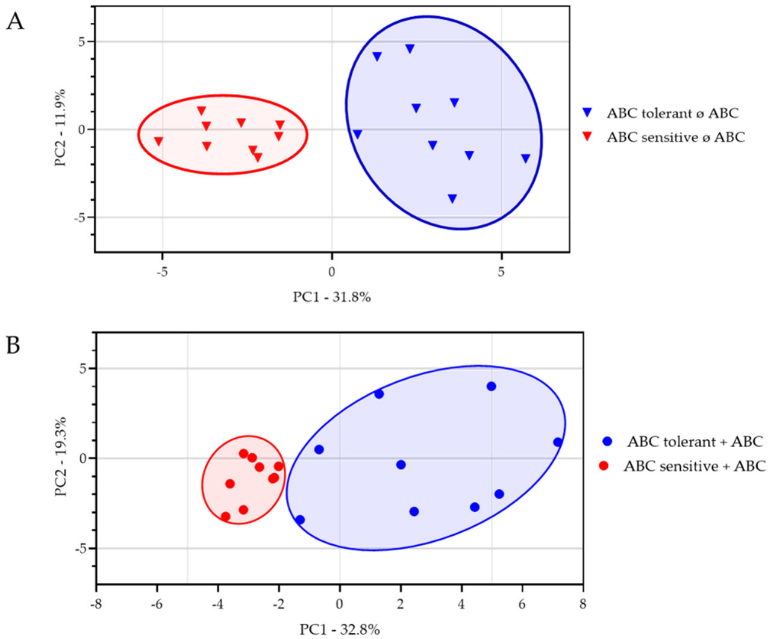

Figure S3: Principal component analysis (PCA) of proteins that were significantly altered ($p < 0.05$) in PBMCs of each three classified ABC sensitive (red) and ABC tolerant (blue) HLA-B*57:01+ healthy donors prior to or following ABC treatment. (**A**) PBMCs were cultured without (ø) ABC for 48 h in three biologically and three technically independent replicates (*n* = 9). (**B**) PBMCs were incubated with (+) ABC for 48 h in three biologically and three technically independent replicates (*n* = 9).

Table S1. Strongest downregulated proteins in ABC sensitive compared to ABC tolerant HLA-B*57:01 carrier after ABC treatment.

**Protein Name**	**Gene Code**	**Log_2 Regulation**	***p*-Value**
Synaptogyrin-2	*SYNGR2*	−3.36	0.004
Platelet-activating factor acetylhydrolase IB subunit beta	*PAFAH1B2*	−2.26	0.038
SPARC	*SPARC*	−2.63	0.018
Guanine nucleotide-binding protein G(q) subunit alpha	*GNAQ*	−2.62	0.019
Xaa-Pro dipeptidase	*PEPD*	−2.16	0.046
GTP-binding protein Rheb	*RHEB*	−2.50	0.024
BRO1 domain-containing protein BROX	*BROX*	−2.46	0.025
Consortin	*CNST*	−2.92	0.010
Hepatoma-derived growth factor-related protein 2	*HDGFRP2*	−2.37	0.031
Very-long-chain (3R)-3-hydroxyacyl-CoA dehydratase 2	*HACD2*	−2.38	0.030

Table S2. Strongest downregulated proteins in ABC sensitive compared to ABC tolerant HLA-B*57:01 carrier without ABC treatment.

**Protein Name**	**Gene Code**	**Log_2 Regulation**	***p*-Value**
Junction plakoglobin	*JUP*	−2.62	0.001
Serpin B3	*SERPINB3*	−2.58	0.005
Desmoglein-1	*DSG1*	−1.82	0.009
Galectin-10	*CLC*	−1.81	0.001
Protein kish-A	*TMEM167A*	−1.80	<0.001
Stomatin-like protein 2, mitochondrial	*STOML2*	−1.61	<0.001
Deoxyuridine 5-triphosphate nucleotidohydrolase, mitochondrial	*DUT*	−1.50	<0.001
Mitochondrial import inner membrane translocase subunit Tim9	*TIMM9*	−1.49	<0.001
Protein Shroom3	*SHROOM3*	−1.45	<0.001
60S ribosomal protein L26	*RPL26*	−1.42	0.004
